# A six-months study on Black Soldier Fly (*Hermetia illucens*) based diets in zebrafish

**DOI:** 10.1038/s41598-019-45172-5

**Published:** 2019-06-13

**Authors:** Matteo Zarantoniello, Basilio Randazzo, Cristina Truzzi, Elisabetta Giorgini, Claudia Marcellucci, Jorge Arturo Vargas-Abúndez, Andrea Zimbelli, Anna Annibaldi, Giuliana Parisi, Francesca Tulli, Paola Riolo, Ike Olivotto

**Affiliations:** 1 Dipartimento di Scienze della Vita e dell’Ambiente, Università Politecnica delle Marche, via Brecce Bianche, 60131 Ancona, Italy; 2 Dipartimento di Scienze delle Produzioni Agroalimentari e dell’Ambiente (DISPAA), Università di Firenze, via delle Cascine 5, 50144 Firenze, Italy; 3 Dipartimento di Scienze Agroalimentari, Ambientali e Animali (Di4A), Università di Udine, via Sondrio, 2, 33100 Udine, Italy; 4 Dipartimento di Scienze Agrarie, Alimentari ed Ambientali, Università Politecnica delle Marche, via Brecce Bianche, 60131 Ancona, Italy

**Keywords:** Physiology, Metabolism

## Abstract

Intensive fish farming relies on the use of feeds based on fish meal and oil as optimal ingredients; however, further development of the aquaculture sector needs new, nutritious and sustainable ingredients. According to the concept of circular economy, insects represent good candidates as aquafeed ingredients since they can be cultured through environmental-friendly, cost-effective farming processes, on by-products/wastes, and many studies have recently been published about their inclusion in fish feed. However, information about the physiological effects of insect-based diets over the whole life cycle of fish is presently missing. At this regard, the present study investigated, for the first time, the effects of Black Soldier Fly based diets (25 and 50% fish meal substitution) administration for a six months period in zebrafish (*Danio rerio*), from larvae to adults. A multidisciplinary approach, including biometric, biochemical, histological, spectroscopic and molecular analyses was applied. Aside a general reduction in fish growth and lipid steatosis, six-months feeding on Black Soldier Fly based diets did not show major negative effects on zebrafish. Gut histological analysis on intestine samples did not show signs of inflammation and both stress markers and immune response markers did not show significant differences among the experimental groups.

## Introduction

The growing development of a modern aquaculture is strictly connected to a continuous search for sustainable feed ingredients able to promote optimal fish growth and welfare. Aquafeeds have been for a long time based on fish meal (FM) and fish oil (FO)^1^. Although these ingredients represent the ideal feed components for fish, they are expensive and often in low supply^2^. Consequently, several different alternative ingredients, in particular of plant origin^3–5^, have been investigated, and some of them are currently used in aquafeed^6^. Nevertheless, no one of these alternatives is able to perfectly replace FM or FO due to their inadequate protein and lipid quantity and quality, unbalanced amino acid profile, poor protein digestibility and/or the presence of anti-nutritional factors^7^. Recently, insects have received great attention as a new ingredient for aquafeed^8^ since they show many advantages like a low environmental impact, the ability to grow on waste and by-products, a high feed conversion efficiency and a low risk of transmitting zoonotic infections^9–11^. Furthermore, as reported in several reviews on the use of insects in aquafeed^12,13^, insects are characterized by high quantity (60–80%) and quality of protein and are rich in essential amino acids. In particular, Diptera larvae show an essential amino acid composition similar to that of FM^14^. However, insects are known to have some critical aspects for fish nutrition such as an unbalanced fatty acid profile [rich in saturated fatty acids (SFA) rather than in polyunsaturated fatty acids (PUFA)] and the presence of chitin^15^, characteristics that might affect fish growth and welfare, especially when high inclusion levels are used^16–18^.

Among approximately one million known insect species, the Black Soldier Fly (BSF) (*Hermetia illucens*) is receiving a growing attention in feed formulation. Several studies have been published over the last years about its inclusion in aquafeeds, but results are still controversial and exclusively focused on a short part of fish life cycle. Some studies were performed during the larval/juvenile phase^11,16,19,20^ while others during the growth-out phase^9,21,22^. Information about the effects of insect-based diets over the whole life cycle of fish is presently missing. For this reason, the present study investigated, for the first time, the effects of BSF-based diets administration, from larvae to adults in zebrafish (*Danio rerio*). In the present study, emphasis has been given to the adult stage since information about the larval responses to the same diets has recently been published^11^. Compared to conventional aquaculture species like trout, turbot, salmon, gilthead seabream and European seabass, zebrafish has the advantage to have a short life cycle (from embryo to adult in about six months) and to provide abundant biological information from genomic sequencing^23–27^.

The present work represents the first comprehensive multidisciplinary study integrating biometric, histological, molecular, gas chromatographic and spectroscopic analyses, in order to better evaluate the biological and physiological responses to the inclusion of insect meal in aquafeeds during the whole zebrafish life cycle.

## Results

### Growth and survival

Fish were divided in three experimental groups: Control group fed fish meal/fish oil diet; Group A fed the diet including 25% BSF full-fat prepupae meal; Group B fed the diet including 50% of BSF full-fat prepupae meal. As concerns standard length, both Group A (27.6 ± 2.0 mm) and Group B (26.4 ± 1.6 mm) were significantly (p < 0.05) smaller than Control (29.8 ± 1.9 mm). Considering wet weight, Group B (0.21 ± 0.04 g) showed a significant lower value (p < 0.05) than Control (0.28 ± 0.03 g), while no significant differences (p > 0.05) were observed between Control and Group A (0.25 ± 0.04 g). Considering survival at six months, no significant differences (p > 0.05) were observed among the experimental groups. Group A reached the highest survival value (65 ± 11%), while Control and Group B showed a 60 ± 9 and 58 ± 7% survival, respectively.

### Fatty acid content and composition

#### Diets

**Figure 1:**
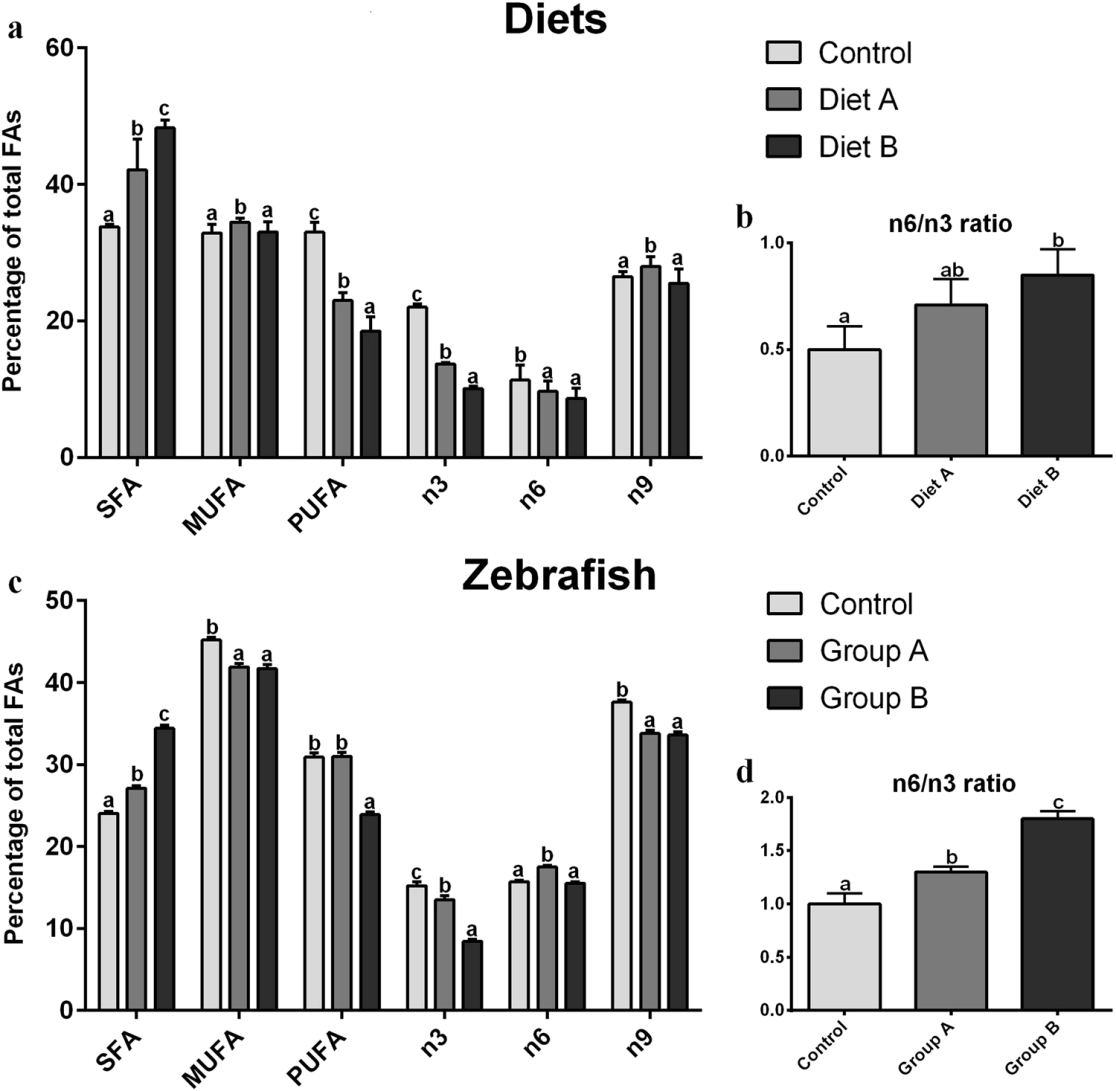
Fatty acids content (as % of total FAs) and n6/n3 ratio. (**a,b**) experimental diets; (**c,d**) adult zebrafish. Control diet was based on fish meal, while A and B diets were characterized by 25 or 50% replacement of fish meal with BSF meal, respectively. Different letters indicate statistically significant differences among experimental groups compared within the same fatty acid class (p < 0.05). Values are presented as mean ± SD (n = 15).

The FAs content (as % of total FAs) of the three experimental diets is presented in Fig. 1a. The increasing inclusion levels of BSF full-fat prepupae meal in the diets resulted in a statistically significant increase (p < 0.05) of SFA content (33.8 ± 0.4, 42.1 ± 4.5 and 48.2 ± 1.2% for Control, A and B diets, respectively) and a parallel decrease in PUFAs content (33.0 ± 1.4, 23.0 ± 1.1 and 18.5 ± 2.1% for Control, A and B diets, respectively; p < 0.05). In particular, as concern both n3 and n6 contents, Control diet (22.0 ± 0.4 and 11.3 ± 2.2%, respectively) showed a significantly (p < 0.05) higher percentage with respect to diet A (13.7 ± 0.2 and 9.7 ± 1.6%, respectively) and diet B (10.1 ± 0.3 and 8.6 ± 1.5%, respectively). Differently, considering monounsaturated fatty acids (MUFA) first and n9 content secondly, no significant differences (p > 0.05) were detected between Control (32.9 ± 1.2 and 26.5 ± 0.7%, respectively) and B diets (33.0 ± 1.4 and 25.5 ± 2.1%, respectively), while diet A (34.5 ± 0.6 and 28.0 ± 1.4%, respectively) was characterized by a significantly (p < 0.05) higher content with respect to the other diets. Finally, no significant differences (p > 0.05) were observed for n6/n3 ratio (Fig. 1b) between Control diet (0.5 ± 0.1) and A diet (0.7 ± 0.1), while B diet (0.8 ± 0.1) showed a significantly (p < 0.05) higher value than Control group. As regards specific FAs composition (% FAMEs; Table 1), the most relevant SFA in all the experimental diets was the palmitic acid (16:0), while lauric acid (12:0) increased significantly according to the dietary BSF meal inclusion. Oleic acid (18:1n9) was the most abundant MUFA in all the dietary treatments. Finally, Control diet showed the highest amount of DHA (22:6n3; 22.2 ± 0.9%) and EPA (20:5n3; 11.3 ± 0.2%) which significantly decreased in diet A (8.2 ± 0.4 and 4.2 ± 0.5%, respectively) and B (6.0 ± 0.7 and 3.1 ± 0.7%, respectively).

**Table 1:** Fatty acid composition (% fatty acid methyl esters) of experimental diets and adult zebrafish.

	Diets			Zebrafish		
	Control	Group A	Group B	Control	Group A	Group B
10:0	n.d.	0.4 ± 0.1^a^	0.7 ± 0.1^a^	n.d.	0.05 ± 0.01^a^	0.07 ± 0.01^b^
12:0	0.1 ± 0.1^a^	6.5 ± 0.7^b^	13.0 ± 1.4^c^	0.3 ± 0.1^a^	5.5 ± 0.4^b^	7.7 ± 0.2^c^
13:0	0.05 ± 0.02^a^	0.04 ± 0.05^a^	0.07 ± 0.04^a^	0.02 ± 0.01^a^	0.02 ± 0.01^a^	0.03 ± 0.01^b^
14:0	4.3 ± 0.2^a^	4.0 ± 0.3^a^	5.7 ± 1.0^b^	2.0 ± 0.1^a^	3.4 ± 0.2^b^	4.7 ± 0.5^c^
14:1n5	n.d.	n.d.	n.d.	0.2 ± 0.1^a^	0.5 ± 0.1^b^	1.0 ± 0.1^c^
15:0	0.7 ± 0.1^a^	0.4 ± 0.1^a^	0.5 ± 0.3^a^	0.4 ± 0.1^a^	0.4 ± 0.1^a^	0.4 ± 0.1^a^
15:1n5	n.d.	n.d.	n.d.	0.2 ± 0.1^a^	0.2 ± 0.1^a^	0.3 ± 0.1^b^
16:0	15.0 ± 1.6^a^	24.1 ± 2.8^b^	21.6 ± 1.2^b^	17.1 ± 0.4^b^	14.7 ± 0.5^a^	17.7 ± 0.4^b^
16:1n9	0.2 ± 0.1^a^	0.1 ± 0.1^a^	0.2 ± 0.1^a^	1.2 ± 0.1^a^	1.4 ± 0.1^a^	1.4 ± 0.2^a^
16:1n7	5.3 ± 0.4^b^	3.9 ± 0.9^a^	5.2 ± 0.8^b^	4.0 ± 0.2^a^	4.4 ± 0.2^a^	4.3 ± 0.2^a^
17:0	0.8 ± 0.1^a^	0.5 ± 0.1^a^	0.6 ± 0.1^a^	0.5 ± 0.1^a^	0.4 ± 0.1^a^	0.4 ± 0.1^a^
17:1n7	n.d.	n.d.	n.d.	0.5 ± 0.1^a^	0.5 ± 0.1^a^	0.5 ± 0.1^a^
18:0	4.9 ± 0.7^a^	5.4 ± 1.1^a^	5.1 ± 0.2^a^	3.5 ± 0.2^b^	2.4 ± 0.1^a^	3.2 ± 0.3^b^
18:1n9	13.2 ± 0.5^a^	27.8 ± 1.6^b^	25.00 ± 1.8^b^	33.6 ± 1.7^a^	30.4 ± 1.3^a^	30.7 ± 1.1^a^
18:1n7	2.1 ± 0.4^b^	1.0 ± 0.2^a^	1.0 ± 0.3^a^	2.8 ± 0.1^c^	2.4 ± 0.2^b^	2.0 ± 0.1^a^
18:2n6	11.2 ± 1.5^b^	8.9 ± 1.5^a,b^	8.0 ± 1.5^a^	12.6 ± 0.7^a^	14.3 ± 0.4^b^	12.2 ± 0.1^a^
18:3n6	0.2 ± 0.03^b^	0.07 ± 0.04^a,b^	0.05 ± 0.06^a^	0.3 ± 0.1^a^	0.3 ± 0.1^a^	0.3 ± 0.1^a^
18:3n3	2.4 ± 0.1^b^	1.1 ± 0.2^a^	0.9 ± 0.3^a^	3.0 ± 0.1^b^	2.6 ± 0.3^b^	1.9 ± 0.3^a^
20:0	0.4 ± 0.1^a^	0.4 ± 0.1^a^	0.4 ± 0.1^a^	0.1 ± 0.1^a^	0.1 ± 0.1^a^	0.1 ± 0.1^a^
20:1n9	1.4 ± 0.3^a^	0.9 ± 0.2^a^	0.9 ± 0.2^a^	2.0 ± 0.1^c^	1.4 ± 0.1^b^	1.1 ± 0.1^a^
20:2n6	0.9 ± 0.1^b^	0.1 ± 0.1^a^	0.1 ± 0.1^a^	0.9 ± 0.1^a^	0.7 ± 0.1^a^	0.7 ± 0.1^a^
20:3n6	0.20 ± 0.04^b^	0.07 ± 0.08^a^	0.05 ± 0.03^a^	0.8 ± 0.1^a^	0.9 ± 0.1^a^	1.0 ± 0.2^a^
21:0	0.07 ± 0.05^a^	0.03 ± 0.03^a^	0.02 ± 0.02^a^	0.01 ± 0.01^a^	0.02 ± 0.01^a^	0.01 ± 0.01^a^
20:4n6	1.2 ± 0.1^b^	0.5 ± 0.1^a^	0.4 ± 0.1^a^	1.2 ± 0.2^a^	1.4 ± 0.1^a^	1.3 ± 0.4^a^
20:3n3	0.2 ± 0.1^a^	0.1 ± 0.1^a^	0.1 ± 0.1^a^	0.4 ± 0.1^b^	0.2 ± 0.1^a^	0.2 ± 0.1^a^
20:5n3	11.3 ± 0.2^b^	4.2 ± 0.5^a^	3.1 ± 0.7^a^	3.2 ± 0.5^c^	2.2 ± 0.3^b^	1.2 ± 0.1^a^
22:0	0.3 ± 0.1^a^	0.3 ± 0.1^a^	0.4 ± 0.1^a^	0.08 ± 0.01^b^	0.08 ± 0.01^b^	0.05 ± 0.01^a^
22:1n9	0.9 ± 0.1^b^	0.4 ± 0.1^a^	0.3 ± 0.1^a^	0.7 ± 0.1^b^	0.5 ± 0.1^a,b^	0.3 ± 0.1^a^
24:0	0.01 ± 0.01^a^	0.05 ± 0.02^a^	0.04 ± 0.05^a^	n.d.	n.d.	n.d.
22:6n3	22.2 ± 0.9^c^	8.2 ± 0.4^b^	6.0 ± 0.7^a^	8.6 ± 0.2^b^	8.5 ± 0.6^b^	5.1 ± 0.1^a^
24:1n9	0.8 ± 0.4^a^	0.4 ± 0.1^a^	0.4 ± 0.1^a^	0.2 ± 0.1^a^	0.1 ± 0.1^a^	0.1 ± 0.1^a^

For each matrix, mean within rows bearing different letters are significantly different (p < 0.05; n = 15). **Diets**: Control diet was based on fish meal, while A and B diets were characterized by 25 or 50% replacement of fish meal with BSF meal, respectively. **Zebrafish**: fish fed diet based on fish meal (Control) and diets with 25% (Group A) or 50% (Group B) replacement of fish meal with BSF meal.

#### Zebrafish

Figure 1c reports the FA content (as % of total FAs) of adult zebrafish fed the different diets. The SFA content showed statistically significant differences (p < 0.05) among experimental groups, increasing from Control (24.0 ± 0.3%) to Group A (27.1 ± 0.3%) and to Group B (34.4 ± 0.4%). Regarding MUFA and n9 contents, both Group A (41.9 ± 0.4 and 33.8 ± 0.4%, respectively) and B (41.7 ± 0.5 and 33.6 ± 0.4%, respectively) showed a significantly lower (p < 0.05) percentage respect to Control zebrafish (45.2 ± 0.3 and 37.6 ± 0.3%, respectively). However, no significant differences were detected between group A and B (p > 0.05). Group B zebrafish showed a significantly (p < 0.05) lower PUFA percentage (23.9 ± 0.3%) both respect to Control (30.9 ± 0.5%) and Group A (31.0 ± 0.5). Considering n6 percentage, no statistically significant differences (p > 0.05) were observed between Control (15.7 ± 0.2%) and Group B (15.5 ± 0.2%), while Group A zebrafish (17.5 ± 0.2%) showed a significantly (p < 0.05) higher percentage than the other groups. Finally, the increasing inclusion levels of BSF full-fat prepupae meal resulted in a statistically significant (p < 0.05) decrease in n3 percentage (15.2 ± 0.5, 13.5 ± 0.5 and 8.4 ± 0.3% for Control, Group A and Group B, respectively) and in a statistically significant (p < 0.05) increase in n6/n3 ratio (1.0 ± 0.1, 1.3 ± 0.1 and 1.8 ± 0.1 for Control, Group A and Group B, respectively; Fig. 1d).

As concern the FAs composition of adult zebrafish (Table 1), the most represented SFAs in all the experimental groups were palmitic acid (16:0) and stearic acid (18:0). Furthermore, the content of lauric (12:0) and myristic (14:0) acids significantly (p < 0.05) increased according to the increasing dietary BSF meal inclusion. Considering MUFA, the predominant fatty acid in all the experimental groups was oleic acid (18:1n9) which did not show statistically significant differences (p > 0.05) among the experimental groups. Linoleic acid (18:2n6) was the most abundant PUFA in all the dietary treatments. In addition, Control group was characterized by a significantly (p < 0.05) higher percentage of EPA (20:5n3; 3.2 ± 0.5%) with respect to Group A (2.2 ± 0.3%) and Group B (1.2 ± 0.1%). On the other hand, Group B (5.1 ± 0.1%) showed a significantly (p < 0.05) lower value of DHA (22:6n3) respect to both Group A (8.5 ± 0.6%) and Control (8.6 ± 0.2%).

### Histology

**Figure 2:**
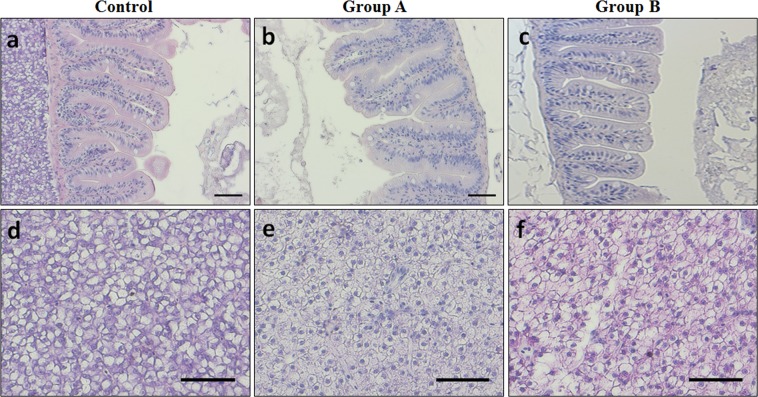
Example of histomorphology of adult zebrafish. (**a–c**) Intestine; (**d–f**) liver. Zebrafish fed diet based on fish meal (Control) and diets with 25% (Group A) or 50% (Group B) replacement of fish meal with BSF meal. Scale bars: **(a–c)** 50 µm; **(d–f)** 100 µm.

Histological analyses were performed on intestine and liver samples and results varied among the experimental groups. All fish, regardless of the diet, did not show any morphological alterations of the intestine (Fig. 2a–c).

As concern liver (Fig. 2d–f), results evidenced a variable degree of lipid accumulation in the experimental groups. A moderate intracytoplasmic lipid accumulation was observed in liver from Control group, characterized by a diffuse presence of hepatocytes with cytoplasm filled of fat, interspersed with normal hepatocytes (Fig. 2d). All liver samples from Group A (Fig. 2e) and, in particular, Group B (Fig. 2f) showed moderate degree of steatosis with swollen hepatocytes and abundant intracytoplasmic lipid accumulation.

### Fourier transform-infrared microspectroscopy (FTIRM) analysis

**Figure 3:**
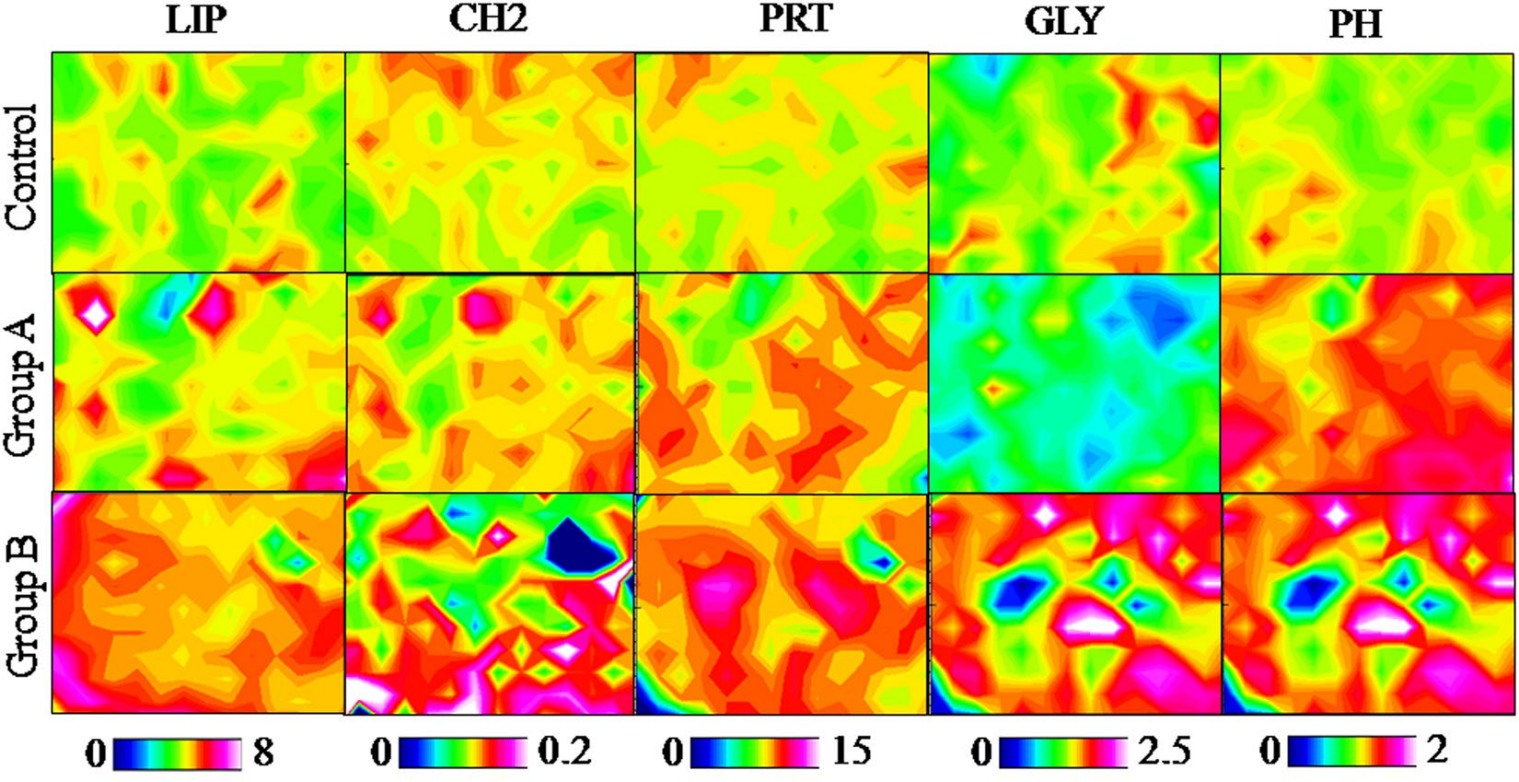
Example of imaging vibrational analysis of Control, Group A and Group B zebrafish liver samples. Topographical distribution of: lipids (LIP); saturated alkyl chains (CH2); proteins (PRT); glycogen (GLY) and phosphate groups (PH). Map size ~560 × 440 µm^2^. Zebrafish fed diet based on fish meal (Control) and diets with 25% (Group A) or 50% (Group B) replacement of fish meal with BSF meal.

The imaging vibrational analysis of three sections representative of Control, Group A and Group B zebrafish liver samples is reported in Fig. 3. With respect to Control liver samples, major amounts of lipids (LIP), saturated alkyl chains (CH2), proteins (PRT), glycogen (GLY) and phosphate groups (PH) were observed in Group B, while an intermediate result was detected in Group A samples.

**Figure 4:**
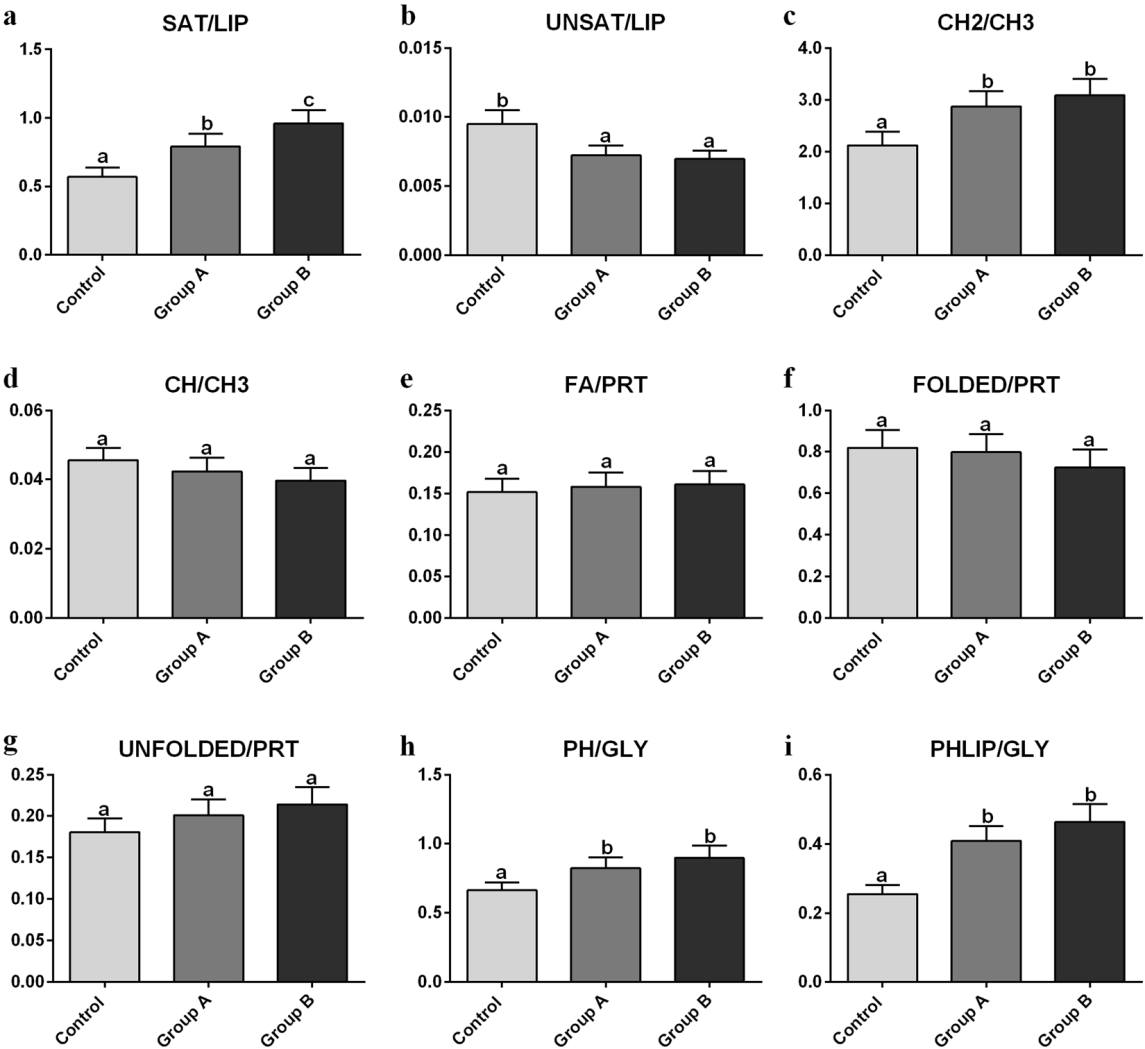
Semiquantitative analysis of the biochemical composition of Control, Group A and Group B liver samples. Statistical analysis of the numerical variation of the following band area ratios: **(a)** SAT/LIP; **(b)** UNSAT/LIP; **(c)** CH2/CH3; **(d)** CH/CH3; **(e)** FA/PRT; **(f)** FOLDED/PRT; **(g)** UNFOLDED/PRT; **(h)** PH/GLY, and **(i)** PHLIP/GLY. Different letters indicate statistically significant differences among experimental groups. Values are presented as mean ± SD (n = 15). Zebrafish fed diet based on fish meal (Control) and zebrafish fed diets with 25% (Group A) or 50% (Group B) replacement of fish meal with BSF meal.

Due to the complexity of the spectral profile and the presence of several convoluted bands, a semiquantitative analysis of the spectral data was performed. At this purpose, for each experimental group, the absorbance average spectrum, together with its standard deviation spectra (absorbance average spectra ± standard deviation spectra) were calculated and curve fitted in the following spectral ranges: 3050–2820 cm^−1^ (containing the vibrational modes of CH, CH2 and CH3 groups of lipid alkyl chains, and hence representative of lipids), 1790–1480 cm^−1^ (containing the vibrational modes related to proteins secondary structure, and hence representative of proteins), and 1280–1000 cm^−1^ (containing the vibrational modes of carbohydrates and phosphates, and hence representative of glycogen and phospholipids). The integrated areas of specific underlying bands with biological meaning were used to calculate the following band area ratios (Fig. 4): SAT/LIP (relative amount of saturated alkyl chains in lipids); UNSAT/LIP (relative amount of unsaturated alkyl chains in lipids); CH2/CH3 (degree of saturation and length of lipid alkyl chains); CH/CH3 (degree of unsaturation of lipid alkyl chains); FOLDED/PRT (relative amount of folded structures in proteins); UNFOLDED/PRT (relative amount of unfolded structures in proteins); FA/PRT (relative amount of fatty acids compared to proteins); PH/GLY (relative amount of phosphate groups compared to glycogen), and PHLIP/GLY (relative amount of phospholipids compared to glycogen). By comparing the numerical variation of the above cited band ratios for Control, Group A and Group B liver samples, the following observations can be made: (i) a higher amount of saturated lipid alkyl chains (SAT/LIP and CH2/CH3; Fig. 4a,c) and a lower quantity of unsaturated ones (UNSAT/LIP and CH/CH3; Fig. 4b,d) were observed in Group A and Group B liver samples with respect to Control one; (ii) no statistically significant variation was detected in the relative amount of fatty acids, with respect to proteins (FA/PRT; Fig. 4e) by comparing all the experimental groups; (iii) regards proteins, a decrease of folded structures (FOLDED/PRT; Fig. 4f) and an increase of unfolded ones (UNFOLDED/PRT; Fig. 4g) was detected (even if both not statistically meaningful) in Group A and Group B liver samples; (iv) a higher amount of phosphate groups (PH/GLY; Fig. 4h) and phospholipids (PHLIP/GLY; Fig. 4i) with respect to glycogen was noticed.

### Real-time PCR results

Real-time PCR analyses were performed on liver samples in order to test the expression of genes involved in fish growth (*igf1*, *igf2a* and *mstnb*), stress response (*nr3c1* and *hsp70.1*) and long-chain polyunsaturated fatty acids biosynthesis (*elovl2, elovl5* and *fads2*). Differently, genes involved in immune response (*il1b, il6* and *tnfa*) and enzymatic hydrolysis of chitin (*chia.2*, *chia.3* and *chia.5*) were investigated in intestine samples.

#### Growth factors

**Figure 5:**
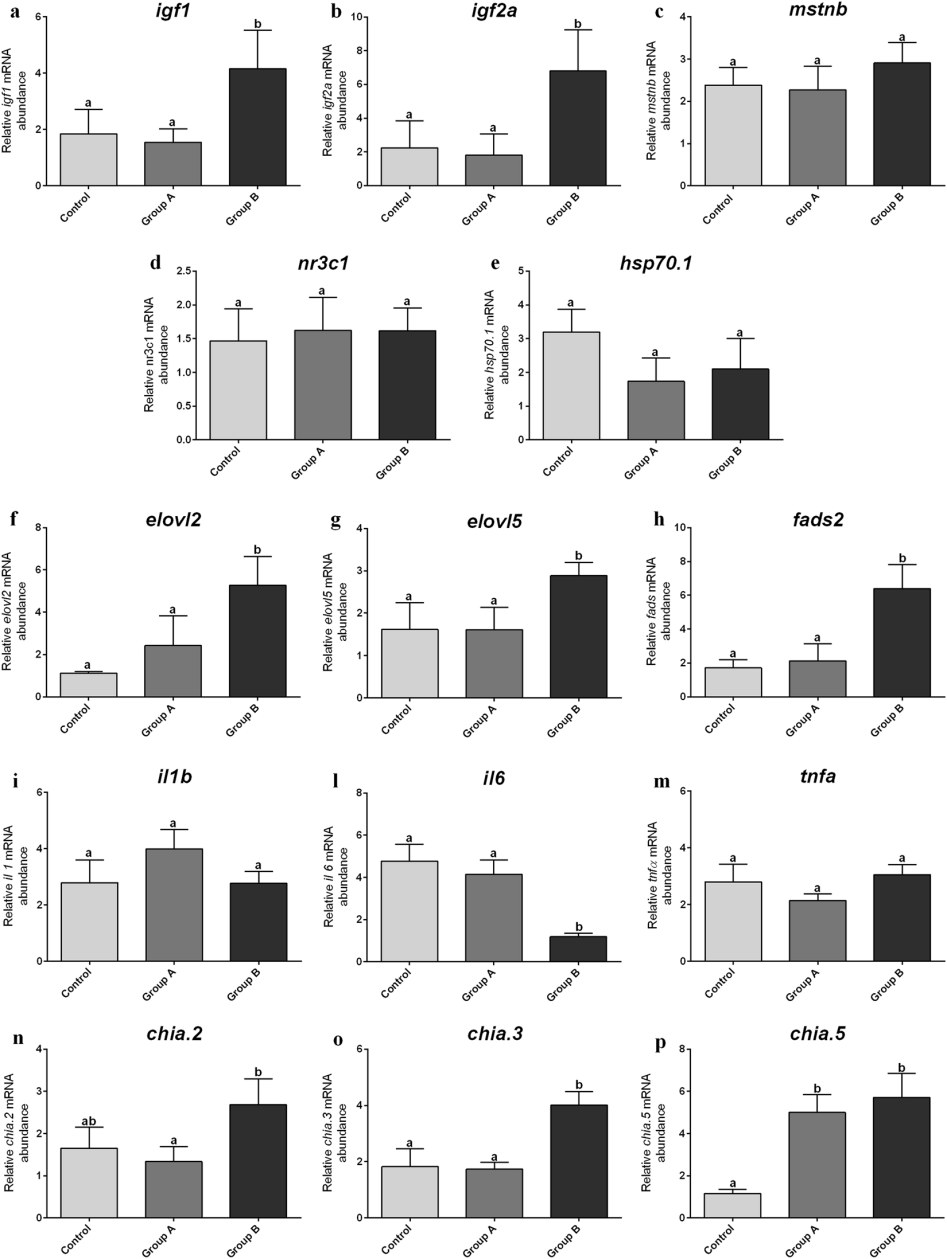
Relative mRNA levels of genes analyzed in adult zebrafish. (**a–c**) growth; **(d,e**) stress response; **(f–h**) lipid metabolism and long-chain polyunsaturated fatty acids biosynthesis; **(i–m**) immune response; **(n–p**) enzymatic hydrolysis of chitin. Zebrafish fed diet based on fish meal (Control) and diets with 25% (Group A) or 50% (Group B) replacement of fish meal with BSF meal. Different letters indicate statistically significant differences among experimental groups (p < 0.05). Values are presented as mean ± SD (n = 15).

Considering both *igfs* gene expression (Fig. 5a,b), no significant differences (p > 0.05) were detected between Control and Group A, while Group B showed significantly (p < 0.05) higher values with respect to both Control and Group A. Differently, regarding *mstnb* gene expression (Fig. 5c), no significant differences (p > 0.05) were observed among all the experimental groups.

#### Stress response

As concerns both *nr3c1* and *hsp70.1* gene expression (Fig. 5d,e), no significant differences (p > 0.05) were evident among the experimental groups.

#### Lipid metabolism

The expression of genes involved in long-chain polyunsaturated fatty acid elongation (*elovl2* and *elovl5*) and desaturation (*fads2*) evidenced a similar pattern (Fig. 5f-h). In particular, the higher the BSF meal inclusion level the higher was the *elovl2*, *elovl5* and *fads2* gene expression. No significant differences (p > 0.05) were detected between Control and Group A.

#### Immune response

Considering *il1b* and *tnfa* gene expression (Fig. 5i,m), no significant differences (p > 0.05) were observed among the experimental groups, while, as concerns *il6* gene expression (Fig. 5l), both Control and Group A showed a significantly (p < 0.05) higher gene expression respect to Group B.

#### Chitinases

Considering *chia.2* and *chia.3* gene expression (Fig. 5n,o), no significant differences (p > 0.05) were evident between Control and Group A, while Group B was characterized by a significantly (p < 0.05) higher gene expression compared to both Group A and, only for *chia 3* gene expression, Control. Finally, as reported in Fig. 5p, expression of *chia.5* was significantly (p < 0.05) higher in Group A and Group B with respect to Control.

## Discussion

With the growing understanding of the role of bio-economy within Europe and the need to apply circularity in the use of natural resources, recognizing that the waste can represent a new starting material for other industrial processes is now a must. In this sense, as suggested by the EU community, a circular economy should be applied also to the aquaculture sector, improving economic benefits, contributing to innovation and growth, encouraging sustainability and competitiveness in the long term.

According to the concept of circular economy, insects represent good candidates as aquafeed ingredients, since they can be cultured through environmental-friendly, cost-effective farming processes, on by-products/wastes^28,29^. In particular, *Hermetia illucens* is one of the most promising insect species especially because of its essential amino acid pattern, similar to that of FM^30–32^. The inclusion of BSF meal in aquafeed has been extensively investigated in the last years but results are still controversial. In addition, all the studies so far performed are focused on a short period of fish life cycle (larval stage, juvenile stage or growth-out stage^16,21,33–36^. To the best of our knowledge, no study about the effects of insect-based diets during the whole life cycle of fish is presently available. One of the possible reasons of this lack of information is mainly related to the fact that most aquaculture species have long life cycles, making these feeding trials expensive and laborious.

At this regard, the use of species that are amenable to experimental investigation provides tools to approach questions that would not be feasible in other ‘non-model’ organisms. For example, small teleost fish such as zebrafish and medaka have the same or similar developmental mechanisms, morphological features and physiological responses of many aquaculture species^37^ providing the ability to use comparative analyses between different organisms to understand mechanisms of development, physiology and possibly providing useful information for finfish production. For these reasons, the present study represents the first report about a six-months feeding trial based on BSF diets on an experimental model, zebrafish.

As reported in previous studies, insect meal could reduce fish growth and welfare especially over longer periods of time and when high percentages of inclusion (more than 25–30%) are used^16,38,39^. At this regard, the present six months experiment evidenced that, even if the tested diets were isoenergetics, fish growth was negatively affected by the increasing percentage of BSF meal in the diets. However, biometric results were not fully supported by the molecular ones that evidenced a significantly higher growth factors (*igf1* and *igf2a*) gene expression in fish fed BSF-based diets. Results are not obvious especially because of the pleiotropic nature of this hormones family which is involved in fish growth regulation but also in many other biological processes like DNA synthesis, cartilage sulfation and protein synthesis, spermatogenesis and final oocyte maturation^40^. Fish growth delay is often related to starvation or malnutrition (for example a long-term deficiency of essential nutrients or presence of anti-nutritional factors)^41,42^. In the present study the delay in growth observed in fish fed BSF-based diets respect to control is probably due to an unbalanced fatty acid composition of the diets. In fact, higher BSF meal inclusion levels in the diets, resulted in lower HUFAs and higher SFA content in the diets.

Many freshwater species, including zebrafish, are able to convert shorter-chain precursors in highly unsaturated FAs through the activation of specific elongase and desaturase^41,43^. After a 6 months treatment period, the fish fed insect meal based diets at the highest percentage of inclusion showed higher elongase and desaturase (*elovl2*, *elovl5* and *fads2*) gene expression compared to Control, and GC analyses revealed the presence of HUFAs, underlying the ability of the fish to promote the above mentioned conversions. These biochemical conversions require energy-expenditure by the fish that may thus explain the observed growth delay. Additionally, zebrafish fed BSF-based diets showed a higher accumulation in SFA respect to Control, particularly evident at the hepatic level. The FTIRM analysis on liver samples evidenced an increase of band area ratios related to SFA in fish fed BSF-based diets.

In addition, as already evidenced by previous studies, high SFA, together with a high n-6/n-3 ratio, caused hepatic steatosis in zebrafish specimens fed BSF-based diets^44–46^.

Aside a general reduction in fish growth, six months feeding on BSF-based diets did not show major negative effects on zebrafish. Gut histological analysis on intestine samples did not show signs of inflammation. Furthermore, both stress markers (*nr3c1* and *hsp70.1*) and two of the immune response markers analyzed (*il1b* and *tnfa*) did not show significant differences among the experimental groups.

These results may both be supported by the fact that zebrafish possess specific chitinases able to digest chitin (with a gene expression dependent on BSF meal inclusion level) or by the anti-inflammatory, antibacterial and antiviral properties of medium-chain fatty acids (especially lauric acid, C12) which are particularly abundant in the BSF-based diets used in this study^47–49^.

In conclusion, this is the first report about a six months feeding study on BSF-based diets during the whole life cycle of a teleost fish (from larvae to adults). Results are encouraging, however, for a wider possible application of insect meal in aquaculture, further research is necessary to improve the insects fatty acid composition in order to better meet fish nutritional requirements.

## Methods

### Ethics

All procedures involving animals were conducted in line with the Italian legislation on experimental animals and were approved by the Ethics Committee of the Università Politecnica delle Marche (Ancona, Italy) (84/94-A). All efforts were made to minimize animal suffering by using an anesthetic (MS222; Sigma Aldrich, Saint Louis, Missouri, USA).

### Diets

Three dietary treatments, including increasing levels of full fat Black Soldier Fly (BSF) meal, were tested in the present study. BSF prepupae were purchased from a commercial company (Smart Bugs s.s. Company, Ponzano Veneto, TV, Italy), where the insects were reared on a substrate composed by corn meal and fruit and vegetable mixture (50:50). Once collected, BSF prepupae were frozen (−80 °C), freeze-dried and minced using liquid nitrogen.

The diets were formulated to be isonitrogenous and isolipidic with BSF full-fat prepupae replacing 25% (Group A) or 50% (Group B) of the fish meal/oil of the Control diet, respectively.

All the experimental diets were sieved to obtain a different granulometry as a function of fish size development (as reported in feeding schedule section). Diets (in triplicate subsamples) were analysed for proximate composition and gross energy content measured by an adiabatic bomb calorimeter (IKA C7000, Werke GmbH and Co., Staufen, Germany)^50^. For details see Table 2.

**Table 2:** Ingredient composition, proximate analysis and gross energy content of the test diets.

	Control	Group A	Group B
***Ingredients (g/kg)***			
Fish meal, Chile, super prime	420	315	210
Peas, protein concentrate	55	78	100
*Hermetia illucens* meal	0	105	210
Wheat, gluten meal	55	78	100
Wheat flour	290	268	255
Fish oil	70	40	28
Palm oil	70	75	56
Min. & Vit. Supplement §	20	20	20
Binder	20	20	20
L-methionine	0	1	1
***Proximate composition***			
Dry matter (%)	4.2 ± 0.1	5.5 ± 0.2	5.3 ± 0.4
Crude protein (%)	40.0 ± 0.5	40.2 ± 0.4	41.1 ± 0.1
Crude lipids (%)	18.6 ± 0.1	17.7 ± 0.2	17.0 ± 0.1
Ash (%)	14.2 ± 0.2	14.1 ± 0.3	12.2 ± 0.6
N-free extractive (NFE, %)	23.0 ± 0.3	22.5 ± 0.6	24.4 ± 1.0
Gross Energy (MJ/kg)	22.1 ± 0.1	22.3 ± 0.1	21.3 ± 0.1

Control diet was based on fish meal, while A and B diets were characterized by 25 or 50% replacement of fish meal with BSF meal, respectively. §Composition of mineral mix (g/kg diet): Ca HPO_4_ *2H_2_O, 27.5; K_2_HPO_4_, 19.0; NaCl, 6.1; MgO, 2.0; FeCO_3_, 1.75; KI, 0.15; ZnO, 0.11; MnO, 0.07; CuSO_4_, 0.02; sodium selenite, 0.002. Composition of vitamin mix (mg/kg diet): thiamine HCl, 40; riboflavin, 40; pyridoxine HCl, 40; cyanocobalamin, 0.2; niacin, 300; calcium pantothenate, 100; folic acid, 5; biotin, 3; choline chloride, 5000; myo-inositol, 1000; ascorbic acid, 2000; a-tocopheryl acetate, 250; menadione, 90; vit. A retinyl palmitate, 40,000 IU/kg diet; vit. D3 cholecalciferol, 2500 IU/kg diet.

### Fish

Zebrafish (*Danio rerio*) AB embryos (for details about the strains please visit https://zfin.org/ZDB-GENO-960809-7) were spawned and maintained 48 h in a Tecniplast system (Varese, Italy), subjected to the following conditions: 28 °C, pH 7.0, NO_2_ and NH_3_ concentrations_ < _0.01 mg/L, NO_3_ < 10 mg/L, respectively and photoperiod 12 L/12D. After this first period, embryos were gently collected, counted under a stereomicroscope (Leica Wild M3B, Leica Microsystems, Nussloch, Germany) and randomly divided in three experimental groups (in triplicate) according to the three test diets.

### Experimental design

Zebrafish larvae were initially reared in 9 tanks (20 L, 3 tanks per experimental group with 500 fish per tank, 1500 per dietary group) and fed the three experimental diets in a tank system according to Olivotto *et al*., 2004 and Falcinelli *et al*., 2015^51,52^. After 30 days post spawning (dps), fish of each tank were transferred in bigger tanks (100 L; 9 in total, 3 per each dietary group) equipped with mechanical and biological filtration (Panaque, Rome, Italy) and fed the same diets for 6 months. Adult 6-months-old zebrafish were collected and anesthetized with a lethal dose of MS222 (1 g/L, Sigma Aldrich, Saint Louis, Missouri, USA), counted to estimate survival rate, and the liver, digestive tract and *in toto* fish were sampled and properly stored for further analyses.

### Feeding schedule

Starting from 5 dps to 6 months, fish were fed as follows. Control group: zebrafish fed fish meal/fish oil diet; Group A: zebrafish fed the diet including 25% BSF full-fat prepupae meal; Group B: zebrafish fed the diet including 50% of BSF full-fat prepupae meal. Feed particle sizes were <100 µm from 5 to 15 dps, 101–200 µm from 16 to 30 dps, 201–400 µm from 31 to 60 dps and 401–600 µm from 61 until the end of the experiment.

Zebrafish were fed the experimental diets (2% body weight, BW) twice a day^53^ and, in addition, from 5 to 10 dps, all groups were fed (one feeding in the morning) on the rotifer *Brachionus plicatilis* (5 ind/mL) according to Lawrence *et al*. (2012)^54^.

### Growth and survival

For growth measurements, 20 fish per tank (i.e. 60 per dietary group) were randomly collected at 6 months and individually measured and weighed. The standard length was determined by a sliding calliper (Measy 2000 Typ 5921, Swiss; precision: 0.1 mm) and the weight by an OHAUS Explorer (OHAUS Europe GmbH, Greifensee, Switzerland) analytical balance (precision: 0.1 mg). Survival was evaluated at the end of the experiment (six months) by counting the number of fish respect to the initial larvae.

### Lipid content and fatty acid composition

Whole fish (5 fish per tank, 15 per dietary group) were analyzed for lipid content and fatty acid composition. *In toto* fish were minced, homogenized (homogenizer MZ 4110, DCG Eltronic, Monza, Italy), freeze-dried (Edwards EF4, Crawley, Sussex, England) and lipid extraction was carried out on lyophilized powders following a microwave-assisted extraction (MAE)^55,56^. Fatty acid methyl esters (FAMEs) were prepared according to Truzzi *et al*. (2017)^56^, using the methyl ester of nonadecanoic acid (19:0; Dr. Ehrenstorfer GmbH, Augsburg, Germany) as internal standard. FAMEs were determined by an Agilent-6890 GC System (Milano, Italy) coupled to an Agilent-5973N quadrupole Mass Selective Detector (MSD) (Milano, Italy). A CPS ANALITICA CC-wax-MS (30 m × 0.25 mm ID, 0.25 μm film thickness) capillary column was used to separate FAMEs. Instrumental conditions for the studied matrices were set up, according to Truzzi *et al*. (2017)^56^. For each sample, at least three runs were performed on the GC-MS. The precision of the proposed method evaluated as in Truzzi *et al*. (2014)^57^ and the limits of detection (LOD) and quantification (LOQ) calculated as in Truzzi *et al*. (2014)^58^, were as in Zarantoniello *et al*. (2018)^11^.

### Histology

Intestines and livers collected from 5 different fish specimens for tank (15 per dietary group) were fixed by immersion in Bouin’s solution (Sigma-Aldrich, Milano, Italy) and stored at 4 °C for 24 h. Samples were prepared according to Giorgini *et al*. (2018)^59^ and 5 µm sections were stained with Mayer hematoxylin and eosin Y (Sigma-Aldrich, Milano, Italy). Sections were observed using a Zeiss Axio Imager.A2 (Oberkochen, Germany) microscope; images were acquired by mean of a combined color digital camera Axiocam 503 (Zeiss, Oberkochen, Germany).

### FTIRM analysis

Livers from 5 different fish specimens for tank (15 per dietary group), were quickly dissected and immediately frozen at −80 °C. Then, from the middle part of each sample, three thin sections (10 µm thick) were cut at 100 µm intervals by using a cryomicrotome (Microm HM 505 N, Neuss, Germany) and deposited onto CaF_2_ optical windows (1 mm thick, 13 mm diameter) for FTIRM analysis^59^. IR measurements were performed at room temperature by using a PerkinElmer Spectrum GXI Spectrometer (Waltham, Massachusetts, USA), equipped with a PerkinElmer AutoIMAGE microscope and a photoconductive HgCdTe MCT array detector, operating at liquid nitrogen temperature (Spectrum Image 5.1.0 software package, Perkin Elmer). By means of a microscope television camera, on each section specific areas were selected on which the IR maps (~560 × 440 µm^2^) were acquired in transmission mode in the spectral range 4000 to 800 cm^−1^ with a spectral resolution of 4 cm^−1^. Background spectra were obtained on clean portions of CaF_2_ optical windows. IR maps are false color images representing the topographical distribution of the total intensity of the infrared absorption within the mapped area; they were made up of 154 pixel/spectra with a spatial resolution of 40 × 40 µm^2^. Each IR spectrum was the result of 128 scans. Raw IR maps were corrected for the contributions of carbon dioxide and water vapor and vector normalized on the full frequency range (to avoid artifacts due to local thickness variations).

On each processed IR map, the topographical distribution of lipids, proteins, saturated alkyl chains, phosphate groups and glycogen was obtained by integration under the following spectral regions (OPUS 7.1 software package, Bruker Optics): 3000–2827 cm^−1^ (representative of lipids, LIP); 1700–1481 cm^−1^ (representative of proteins, PRT); 1481–1429 cm^−1^ (representative of saturated alkyl chains, CH2); 1280–1189 cm^−1^ (representative of phosphate groups, PH), and 1066–975 cm^−1^ (representative of glycogen, GLY). An arbitrary color scale was used: white color indicated the pixel with the highest IR absorbance values, while blue color the lowest ones.

For each map, the absorbance average spectrum, together with its standard deviation spectra (absorbance average spectra ± standard deviation spectra) were calculated (OPUS 7.1 software package, Bruker Optics, Billerica, Massachusetts). Spectra were interpolated in the 3050–2820 cm^−1^, 1790–1480 cm^−1^ and 1280–1000 cm^−1^ ranges, straight baseline corrected, vector normalized and then curve fitted by using Gaussian curves (Grams A/I 9.1 software package, Galactic) in the same intervals. The position and the area integrals of all the underlying bands were obtained, and these latter used to calculate specific band area ratios (see Results section).

### RNA extraction and cDNA synthesis

Total RNA extraction from both intestine and liver samples from 5 different specimens from each tank (15 fish per dietary group) was optimized according to Zarantoniello *et al*., (2018)^11^.

### Real-Time PCR

PCRs were performed with SYBER Green in an iQ5 iCycler thermal cycler (both from Bio-Rad) in triplicate. Reactions (10 µL) were run according to Zarantoniello *et al*., (2018)^11^. The thermal profile for all reactions was: 3 min at 95 °C, followed by 45 cycles of 20 s at 95 °C, 20 s at 60 °C and 20 s at 72 °C. Fluorescence was monitored at the end of each cycle. Dissociation curve analysis showed a single pick in all cases.

Relative quantification of the expression of genes analyzed was performed using *arp* and *rpl13* as housekeeping genes to standardize the results (Table 3).

**Table 3:** Primer sequences and the zebrafish information network (zfin) used in this study.

Gene	Forward primer (5′-3′)	Reverse primer (5′-3′)	ZFIN ID
igf1	5′-GGCAAATCTCCACGATCTCTAC-3′	5′-CGGTTTCTCTTGTCTCTCTCAG-3′	ZDB-GENE-010607-2
igf2a	5′-GAGTCCCATCCATTCTGTTG-3′	5′-GTGGATTGGGGTTTGATGTG-3′	ZDB-GENE-991111-3
mstnb	5′-GGACTGGACTGCGATGAG-3′	5′-GATGGGTGTGGGGATACTTC-3′	ZDB-GENE-990415-165
nr3c1	5′-AGACCTTGGTCCCCTTCACT-3′	5′-CGCCTTTAATCATGGGAGAA-3′	ZDB-GENE-050522-503
hsp70.1	5′-TGTTCAGTTCTCTGCCGTTG-3′	5′-AAAGCACTGAGGGACGCTAA-3′	ZDB-GENE-990415-91
elovl2	5′-CACTGGACGAAGTTGGTGAA-3′	5′-GTTGAGGACACACCACCAGA-3′	ZDB-GENE-011212-1
elovl5	5′-TGGATGGGACCGAAATACAT-3′	5′-GTCTCCTCCACTGTGGGTGT-3′	ZDB-GENE-040407-2
fads2	5′-CATCACGCTAAACCCAACA-3′	5′-GGGAGGACCAATGAAGAAGA-3′	ZDB-GENE-011212-1
il1b	5′-GCTGGGGATGTGGACTTC-3′	5′-GTGGATTGGGGTTTGATGTG-3′	ZDB-GENE-040702-2
il6	5′-CTGGAGGCCATAAACAGCCA-3′	5′-TGCGAGTCCATGCGGATTTA-3′	ZDB-GENE-120509-1
tnfa	5′-TTGTGGTGGGGTTTGATG-3′	5′-TTGGGGCATTTTATTTTGTAAG-3′	ZDB-GENE-050317-1
chia.2	5′-GGTGCTCTGCCACCTTGCCTT-3′	5′-GGCATGGTTGATCATGGCGAAAGC-3′	ZDB-GENE-040426-2014
chia.3	5′-TCGACCCTTACCTTTGCACACACCT-3′	5′-ACACCATGATGGAGAACTGTGCCGA-3′	ZDB-GENE-040426-2891
chia.5	5′-CCACGGCTCACAGGACAACATCA-3′	5′-GTCCGCAGACGACAGGCGAA-3′	ZDB-GENE-071004-113
arp	5′-CTGAACATCTCGCCCTTCTC-3′	5′-TAGCCGATCTGCAGACACAC-3′	ZDB-GENE-040116-1
rpl13	5′-TCTGGAGGACTGTAAGAGGTATGC-3′	5′-AGACGCACAATCTTGAGAGCAG-3′	ZDB-GENE-031007-1

Amplification products were sequenced, and homology was verified. No amplification product was detected in negative controls and no primer-dimer formation was found in control templates. Data were analyzed using the iQ5 optical system software version 2.0, including Genex Macro iQ5 Conversion and Genex Macro iQ5 files (all from Bio-Rad). Modification of gene expression was reported with respect to controls. Primer sequences were designed using Primer3 (210 v. 0.4.0) starting from zebrafish sequences available in ZFIN.

### Statistical analysis

All data were analyzed by one-way ANOVA, with diet as the explanatory variable. All ANOVA tests were followed by Tukey’s post-hoc test. The statistical software package Prism5 (GraphPad Software) was used. Significance was set at *p* < 0.05 and all the results are presented as mean ± SD.
